# Divergent influences of the locus coeruleus on migraine pathophysiology

**DOI:** 10.1097/j.pain.0000000000001421

**Published:** 2018-12-14

**Authors:** Marta Vila-Pueyo, Lauren C Strother, Malak Kefel, Peter J. Goadsby, Philip R. Holland

**Affiliations:** a Headache Group, Department of Basic and Clinical Neuroscience, Institute of Psychiatry, Psychology and Neuroscience, King's College London, London, United Kingdom; b NIHR-Wellcome Trust, King's Clinical Research Facility, King's College Hospital, London, United Kingdom

**Keywords:** Migraine, Pain, Fatigue, Locus coeruleus

## Abstract

Migraine is a common disabling neurological condition that is associated with several premonitory symptoms that can occur days before the headache onset. The most commonly reported premonitory symptom is marked fatigue that has been shown to be highly predictive of an ensuing migraine attack. The locus coeruleus (LC) is a key nucleus involved in arousal that has also been shown to impact pain processing. It provides one of the major sources of noradrenaline to the dorsal horn of the spinal cord and neocortex. Given the clinical association between migraine, sleep–wake regulation, and fatigue, we sought to determine whether LC modulation could impact migraine-related phenotypes in several validated preclinical models of migraine. To determine its role in migraine-related pain, we recorded dural nociceptive-evoked responses of neurons in the trigeminocervical complex, which receives trigeminal primary afferents from the durovascular complex. In addition, we explored the susceptibility to cortical spreading depression initiation, the presumed underlying phenomenon of migraine aura. Our experiments reveal a potent role for LC disruption in the differential modulation of migraine-related phenotypes, inhibiting dural-evoked activation of wide dynamic neurons in the trigeminocervical complex while increasing cortical spreading depression susceptibility. This highlights the potential divergent impact of LC disruption in migraine physiology, which may help explain the complex interactions between dysfunctional arousal mechanisms and migraine.

## Abstract 2

Supplemental Digital Content is Available in the Text.

In vivo, electrophysiology highlights a role for the locus coeruleus in trigeminal nociception and migraine aura, linking pain and dysfunctional arousal in migraine.

## 1. Introduction

Migraine ranks the second most common cause of years lost to disability globally.^20^ Despite extensive research and clinical development, there remains a major gap in migraine treatment. To date, most studies have focused on targeting migraine pain^12,15,37^; however, there is an increasing understanding of the importance of premonitory (prodromal) symptoms as the earliest identifiable predictors of an ensuing attack.^7,24^ Although the literature on the epidemiology of premonitory symptoms is not entirely resolved, key neurological symptoms such as fatigue have emerged as highly prevalent and highly predictive attack hallmarks.^11,22,43^ The mechanisms underlying the presence of abnormal fatigue and its relationship with migraine-related nociceptive processing remain enigmatic. Migraine is intrinsically linked with regulation of sleep–wake cycles, with sleep disruption a commonly reported migraine trigger, while sleep itself is commonly reported for attack normalisation.^31^ The wake promoting locus coeruleus (LC) shows clear diurnal activity levels^30^ with increased activity during arousal and almost complete inactivity during sleep. As such, we sought to explore the impact of dysregulation of the LC on migraine-related phenotypes in validated preclinical models.

A potential role for the LC in migraine pathophysiology is supported by both preclinical and clinical evidence. It is responsive to trigeminovascular activation,^50,51^ and several human neuroimaging studies have highlighted altered activity and functional connectivity of the dorsal rostral pons (that contain the LC among other nuclei), in migraine.^2,33,35,44^ Functionally, the LC modulates a wide variety of networks having a key role in arousal, cognition, nociception, and stress circuits^45^ through descending projections to the spinal cord and ascending projections throughout the central nervous system. These ascending and descending projections have been shown to have divergent effects on behaviour, with descending projections largely regulating spinal nociception^27^ and ascending projections involved in multiple processes including nociception, stress responses, aversive behaviours, and cognition.^27,45^

Importantly, the LC may directly modulate spinal trigeminal nucleus neurons^42^ and its stimulation results in α2-adrenoceptor–dependent^25^ cerebral hypoperfusion,^23^ which is a known trigger of cortical spreading depression (CSD),^49^ the presumed underlying phenomenon of migraine aura.^8^ In this study, we characterised the impact of LC modulation in 2 validated animal models of migraine, acute dural-evoked activation of the trigeminovascular system, and the threshold for the induction of CSD. First, we determined the impact of acute and chronic ablation of the LC on trigeminovascular nociceptive responses that are relevant to headache and identified the noradrenergic receptor subtypes responsible for the observed changes. In addition, we explored the impact of chronic LC ablation on the electrical and chemical thresholds for CSD induction. Some data have been presented previously in preliminary form.^53,54^

## 2. Materials and methods

All experiments were conducted in accordance with the UK Home Office Animals (Scientific Procedures) Act 1986 and consistent with the ARRIVE guidelines and the guidelines of the Committee for Research and Ethical Issues of International Association for the Study of Pain.^56^ Male Sprague–Dawley rats (n = 97, 220-380 g; Charles River, United Kingdom) were maintained and group-housed under standard conditions (12-hour light–dark cycles; lights on 07:00) with food and water available ad libitum. All animals were randomly assigned to experimental groups based on appropriate sample size calculations (G*Power^18^), and all analyses were conducted by an observer blinded to the experimental grouping. To avoid confounding effects regarding the diurnal cycle, all experiments were performed between the hours of 10:00 and 15:00.

### 2.1. Chronic ablation of locus coeruleus

A subset of rats (n = 58, 240-300 g) were injected with either 50 mg/kg DSP-4 (N-(2-chloroethyl)-N-ethyl-2-bromobenzylamine hydrochloride; Sigma, Dorset, United Kingdom), a neurotoxin that initially selectively destroys noradrenergic projections and cells from the LC,^40^ or saline intraperitoneally 15 days before surgery. Because of the instability and light sensitivity of DSP-4, dilutions in saline were prepared immediately before injection. DSP-4 was observed to induce a characteristic general decrease in locomotor activity within the first 2 hours after injection, and animals were more lethargic throughout the first 4 days and had a 16% mortality rate within the same period.^48^

### 2.2. In vivo preparation

#### 2.2.1. Common surgery

On the day of the surgery, rats were initially anesthetised with isoflurane (IsoFlo, 5%; Abbott, Maidenhead, United Kingdom) and maintained with intravenous propofol infusion (Propoflo, 33-50 mg/kg/h, Abbott). The left femoral artery and vein were cannulated for blood pressure recordings and infusion of anaesthetic, respectively. A tracheotomy was performed to ventilate the animal with oxygen-enriched air and monitor the end-tidal CO_2_ throughout the experiment to keep within physiological parameters (3.5%-4.5%). A rectal probe connected to a heating pad was used to keep the body temperature constant at 36.5 to 37°C. Rats were placed in a stereotaxic frame and underwent one of the experimental surgical preparations and experimental procedure as detailed below.

After each experiment, animals were euthanized by an overdose of intravenous pentobarbitone (Euthatal, 200 mg/kg; Boehringer Ingelheim Animal Health UK, Berkshire, United Kingdom). When the tissue was needed for immunohistochemistry, the animals were perfused with 300 mL of 0.01-M cooled heparinised phosphate buffer saline (PBS), followed by 250 mL of 4% paraformaldehyde in 0.01-M PBS (pH 7.4). The brain and spinal cord were removed and stored for 1 hour in the same fixative and then placed in a cryoprotectant solution (30% sucrose in 0.01-M PBS) for at least 48 hours before being serially sectioned on a freezing cryostat.

Physiological data for all experiments were displayed and saved on a personal computer using an online data analysis system (Power 1401plus and Spike5 v8.04 software; CED, Cambridge, United Kingdom).

#### 2.2.2. Dural-evoked trigeminal activation in the trigeminocervical complex

A parietal craniotomy provided access to the dura mater overlying the middle meningeal artery, and the area was covered in mineral oil. To access the trigeminocervical complex (TCC), a partial laminectomy of the first cervical vertebra was performed, and the dura mater was opened to expose the caudal medulla. After completion of the surgery, animals were left to stabilize for at least 30 minutes before recording.

Stimulation of perivascular afferents of the trigeminal nerve was performed by placing a bipolar stimulating electrode on the dura mater adjacent to the middle meningeal artery. Dural nociceptive neurons in the TCC were identified through electrical stimulation (8-15 V, 0.5 Hz, 0.3-0.5 ms, and 20 square wave electrical pulses) of the dura mater. Stimulation parameters commonly activated Aδ fibers with latencies between 5- and 20-ms range, and less frequently, C fibers with latencies greater than 20 ms.

Tungsten microelectrodes (0.5-1.5 MΩ) were used to record extracellularly from neurons in the TCC activated by dural electrical stimulation and with cutaneous facial receptive fields in the ophthalmic dermatome. The signal from the recording electrode was fed through an AC preamplifier (Neurolog NL104, gain ×1000), through filters (NL125, bandwidth 300 Hz-20 KHz) and a 50-Hz noise eliminator (Humbug), then to a second-stage amplifier (Neurolog NL106, variable gain ×20 to ×90), a gated amplitude discriminator (Neurolog NL201) and an analogue-to-digital converter (Power 1401plus, CED) connected to a computer where it was processed and stored (Spike5 v8.04 software, CED).

When a cluster of neurons sensitive to stimulation of the ophthalmic dermatome of the trigeminal nerve was identified, it was tested for convergent input from the dura mater. Trains of 20 stimuli were delivered at 5-minute intervals to assess the baseline response to dural electrical stimulation. Responses were analysed using poststimulus histograms with a sweep length of 100 ms and a bin width of 1 ms. When stable baseline values of the stimulus-evoked responses were achieved (average of 3 stimulation series), responses were tested for up to 60 minutes after physiological intervention. After the experiment, animals were euthanized, and electrolytic lesions were performed in the TCC (150 µA, 120 seconds) to confirm the location of the recording electrode.

#### 2.2.3. Acute lesion and drug injection in the locus coeruleus

Rats were placed in a stereotactic frame with the nose tilted down, so that bregma was 2.33 mm below lambda. A craniotomy of approximately 2 × 2 mm was performed on the interparietal bone to gain access to the LC. A concentric bipolar tungsten microelectrode, or a 4-barrelled glass micropipette, was placed in the LC (anterioposterior −3.4, mediolateral −1.3, and dorsoventral −6.25 mm from lambda) for electrolytic lesion (200 µA, 500 µs, 30 Hz during 3 minutes) or for microinjection, respectively. After each experiment, the location of the lesion or microinjection was confirmed by cryosectioning the brainstem.

Yohimbine hydrochloride, an α2 adrenoreceptor antagonist (10 mg/mL), clonidine hydrochloride, an α2 adrenoreceptor agonist (1 or 10 mg/mL), phenylephrine hydrochloride, an α1 adrenoreceptor agonist (10 mg/mL), or Chicago Sky Blue 6B powder (2%) were dissolved in saline and injected at a volume of 210 nL. Doses were chosen based on literature and preliminary studies.^1,26,46,55^

#### 2.2.4. Superior sagittal sinus stimulation

In animals treated with saline or DSP-4, to gain access to the superior sagittal sinus (SSS), the skull was exposed and a craniotomy of the parietal bone was performed with saline-cooled drilling from bregma to lambda. Two platinum hook electrodes were then placed on the dura mater over the SSS. Effort was taken to minimize contact between the cortex and stimulating electrodes to reduce the risk of current spread to the cortex. The area was bathed in warmed mineral oil, and the animals rested for 1 hour to minimize nonspecific c-Fos protein expression. Animals were then randomly divided into 2 experimental groups as follows:

(1) Sham controls: after the 1-hour rest period with the electrodes over the SSS, animals remained in the frame for a further 2 hours, receiving no stimulation or any other manipulation (DSP-4: n = 6; saline: n = 6).
(2) Stimulation: after the 1-hour rest period with the electrodes over the SSS, animals received 2 hours of electrical stimulation (0.5 Hz, 0.5-ms duration at 20-28 V; DSP-4: n = 6; saline: n = 6).

After the experiment, animals were euthanized and perfused as described above.

#### 2.2.5. Cortical spreading depression

In animals treated with saline or DSP-4 (n = 14/group), anterior to lambda, a craniotomy of approximately 2 × 2 mm was performed in each parietal bone using a saline-cooled drill, and dura mater was removed. This area was used for electrical or chemical CSD induction. Posterior to bregma, a similar area was drilled in each parietal bone, and dura mater was also removed. In this area, a glass pipette with a tip diameter of 10 µm filled with 3-M NaCl was placed 500 µm below the cortical surface (layer 4), for cortical steady state potential recording (direct current shift). The saline-filled glass pipette was coupled to a micropipette holder containing an Ag/AgCl pellet to facilitate connection to a high-impedance headstage. An Ag/AgCl reference electrode was placed subcutaneously on the neck for differential recording. The signal was fed through a direct current preamplifier (Neurolog NL102, gain ×1000), through filters (NL125) and a 50-Hz noise eliminator (Humbug), then to a second-stage amplifier (Neurolog NL106) and an analogue-to-digital converter (Power 1401plus, CED) connected to a computer where it was processed and stored (Spike5 v8.04 software, CED).^29^

After 30 minutes of baseline recordings in the left hemisphere, the left cortex was electrically stimulated at 5-minute intervals with a bipolar electrode by increasing the electric charge (5, 12.5, 25, 50, 100, 150, 200, 250, 300, 400, 500, 600, 800, 1000, and 1200 µC) until a CSD was induced. After obtaining the electrical threshold for CSD in the left hemisphere, baseline recordings were performed for 30 minutes in the right hemisphere, followed by chemical induction by placing a cotton ball soaked in 1-M KCl on the cortex surface. Cortical spreading depressions were counted for 1 hour with KCl refreshed every 15 minutes (5 µL). After the experiment, animals were euthanized and perfused as described above. We do not expect an ordering effect of testing CSD susceptibility on ipsilateral and contralateral hemispheres in the same animal because CSDs do not propagate to and have been shown to have little effect on the contralateral hemisphere.^17^ To minimize this, we conducted the electrical induction protocol first because it generates a single CSD wave and as such is the least disruptive to cortical function. Furthermore, to control for a potential ordering effect, the protocol was identical between saline- and DSP-4–treated rats.

### 2.3. Immunohistochemistry

Cryosectioned tissue (30 μm) spanning the TCC (trigeminal nucleus caudalis and cervical spinal cord levels C1 and C2), the LC, and the cortex were collected in an antifreeze solution (30% ethylene glycol, 20% glycerol in 0.01-M PBS) and stored at −20°C until used.

In rats treated with DSP-4 or saline, the effectiveness of DSP-4 treatment was determined by immunoreaction to dopamine-β-hydroxylase (DBH) in the LC, cortex, and TCC. Sections of the LC, cortex, and TCC (n = 2 per animal) were washed, blocked, and incubated with a mouse anti-DBH antibody (1:300, overnight at 4°C; MAB308; Millipore, Watford, United Kingdom). Immunodetection was visualised with a fluorescent goat anti-mouse secondary antibody (1:500, 90 minutes at RT; Alexa Fluor 568, A11031; Thermo Fisher, Dartford, United Kingdom). Sections were washed in 0.01-M PBS, mounted on glass slides, cover-slipped with Vectashield (Vector Laboratories, Peterborough, United Kingdom) and analysed by fluorescent microscopy. Quantification of the expression of DBH was performed by a blinded researcher in ImageJ 1.49v software by measuring the percentage of the area stained, adjusting the same threshold for all the images, and the mean for each animal was calculated (n = 2 sections per animal, bilateral average). Values are expressed as the percentage loss of DBH staining. Saline-treated controls were considered to have 100% staining.

In rats where the SSS was stimulated, neuronal activation of the TCC was assessed by detecting the immunoreactivity to c-Fos in the TCC. Sections of the TCC (n = 12 per animal) were washed, blocked, and incubated with a rabbit anti-c-Fos antibody (1:10,000, overnight at 4°C; ABE457, Millipore). Immunodetection was achieved with a goat anti-rabbit biotinylated secondary antibody (1:500, 90 minutes at RT; BA-1000, Vector Laboratories), followed by an ABC solution amplification (1:200, 30 minutes at RT; ABC Elite Kit, Vector Laboratories) and a colorimetric reaction with 3,3′-diaminobenzidine (DAB) Peroxidase Substrate Kit (1:50, 1-5 min at RT; SK-4100, Vector Laboratories). Sections were washed in 0.01-M PBS, mounted on glass slides, dehydrated, and cover-slipped with DPX (Sigma). The expression of c-Fos was identified by a blinded researcher through the identification of c-Fos–immunoreactive nuclei that were clearly distinguishable from the background level. Quantification of the expression of c-Fos in the TCC (laminae I, II, and V) was performed by 2 independent counters using an optical light microscope. Values are expressed as the median and interquartile ranges per animal.

### 2.4. Statistical analysis

Statistical analysis of raw data was performed using IBM SPSS 22.0 software, and graphs were plotted with SigmaPlot 12.5 software. For each study, n was chosen to obtain a statistical power of 80% to 90%, and analyses of power were performed with G*Power software.^18^ Regarding CSD experiments, animals were included in the analysis only if data from both electrical and chemical stimulation were obtained. For the electrical stimulation, a threshold corresponding to the lowest current needed to induce a CSD was determined for each animal. For chemical stimulation, the number of CSDs where the cortical steady state potential was altered by at least 5 mV was counted over 1 hour.

For CSD experiments and immunohistochemistry, we first tested for normality using the Kolmogorov–Smirnov test. If data were normally distributed, an independent Student *t* test was used for comparison (data expressed as mean ± SEM). If data were not normally distributed, we used Kruskal–Wallis test with Monte Carlo exact test post hoc correction (confidence interval of 95%) and Mann–Whitney *U* test for comparison with Bonferroni post hoc correction (data expressed as a median with interquartile range).

For TCC recordings, data collected of Aδ-fiber activation represent the normalized data for the number of cells firing over a 10-ms period in the region 5 to 20 ms after stimulation over 20 sweeps and are expressed as mean ± SEM. We first analysed whether there was a significant effect over time in the raw data using an analysis of variance for repeated measures with Bonferroni post hoc correction for multiple comparisons; if Mauchly test of sphericity was violated, appropriate corrections to the degrees of freedom were made according to Greenhouse-Geisser.^19^ If the analysis of variance for repeated measures was significant, we proceeded to conduct post hoc analyses. For post hoc analyses, we first compared the similarity of baseline responses across groups. If baseline responses were not different, we compared the effect of the acute LC lesion or the yohimbine, clonidine, or phenylephrine microinjection in the LC at each time point (30 and 60 minutes after lesion; 1, 5, 15, 30, 45, and 60 minutes after injection) with the control group (electrode placed in the LC or saline injection). Data were plotted as percentage of the mean of the 3 baselines obtained either before lesioning or injecting in the LC (normalized data). Animals were only included in the analysis if lesions or microinjections were performed in the LC.

## 3. Results

### 3.1. Locus coeruleus modulation

We applied 3 strategies for modulating the LC. First, to determine the impact of LC disruption, we electrolytically lesioned the LC. Second, to model a more chronic depletion of LC noradrenergic fibres, we used DSP-4, a toxin that is known to ablate selectively LC noradrenergic neurons^40^ because of its uptake through the noradrenaline transporter. Finally, we explored the impact of acute pharmacological modulation of the LC to characterise potential underlying receptor mechanisms.

#### 3.1.1. Acute disruption of the locus coeruleus inhibits dural-evoked trigeminovascular activation

**Figure 1.**
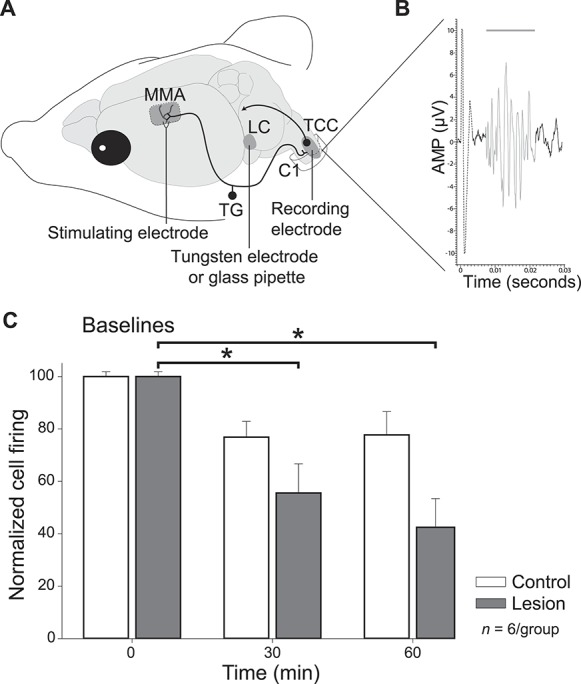
Acute lesion of the locus coeruleus (LC) disrupts trigeminovascular activation in a preclinical migraine model. (A) Schematic representation of the experimental setup of the model of dural-evoked trigeminovascular activation. A bipolar stimulating electrode is placed over the middle meningeal artery (MMA) to induce nociceptive activation of trigeminal afferents that signal through the trigeminal ganglion (TG) and innervate the dural vasculature, while projecting centrally to the trigeminocervical complex (TCC). A recording electrode placed in the TCC and a concentric bipolar tungsten electrode or a glass pipette placed in the LC allow for recording and lesion/microinjection, respectively. (B) An example of TCC response showing neuronal activation (in gray) to dural stimulation (dashed line). (C) Time course changes in the average response of dural-evoked Aδ-fiber trigeminal neurons after placement of an electrode in the LC (control) or electrolytic lesion of the LC. Placement of an electrode in the LC (n = 6) induced a nonsignificant decrease in dural-evoked nociceptive neuronal activation in the TCC (white bars). Subsequent electrolytic lesioning of the LC (n = 6) induced a significant decrease in dural-evoked neuronal activation in the TCC when compared with control (30 minutes: *t*_(5)_ = 2.76, *P* = 0.04; at 60 minutes: *t*_(5)_ = 3.24, *P* = 0.023, gray bars) (AMP, amplitude; C1, first cervical level. Data are expressed as mean ± SEM, *P < 0.05).

To assess the impact of disrupted LC signalling, we electrolytically lesioned the LC in anesthetised rats and monitored the impact of LC disruption on dural-evoked trigeminovascular activation in the TCC, using in vivo extracellular electrophysiology that is a well-established preclinical model of migraine-related nociceptive processing. As illustrated in Figure 1A, electrical stimulation of the perivascular afferents of the trigeminal nerve results in activation of second-order ascending projections from the TCC that can be recorded in vivo (Fig. 1B).

After the establishment of stable dural-evoked wide dynamic range neuronal responses, a concentric bipolar electrode was lowered into the LC in agreement with the atlas of Paxinos and Watson.^38^ Placement of the electrode alone without stimulation induced a stable nonsignificant decrease of TCC dural-evoked neuronal activation (72 ± 11%; *t*_(5)_ = 1.93, *P* = 0.112; Fig. 1C). Subsequent electrolytic lesioning of the LC (pulses of 200 µA, 30 Hz, 500 µs during 3 minutes) resulted in a significant reduction in dural-evoked neuronal activation (F_6,90_ = 4.361, *P* = 0.001), reaching its maximum at 60 minutes after lesion (42 ± 11%; *t*_(5)_ = 3.24, *P* = 0.023; Fig. 1C), indicating a decrease in dural-evoked trigeminal activation. There were no significant differences in baseline spontaneous firing throughout the entire experiment, suggesting the LC effects were nociceptive-specific (F_11,110_ = 0.693, *P* = 0.74). Animals were only included in the analysis if anatomical lesions were located in the LC.

#### 3.1.2. Chronic disruption of the locus coeruleus with DSP-4

Given the antinociceptive effect of acute LC disruption and the previously reported antinociceptive and pronociceptive effects of LC activation,^14,27^ we considered that electrolytic lesioning of the LC may initially result in acute activation of the LC and increased noradrenergic signalling at its terminals. Therefore, to confirm the antinociceptive effect of LC disruption on dural-evoked trigeminovascular activation, we sought to chronically ablate LC noradrenergic neurons.

**Figure 2.**
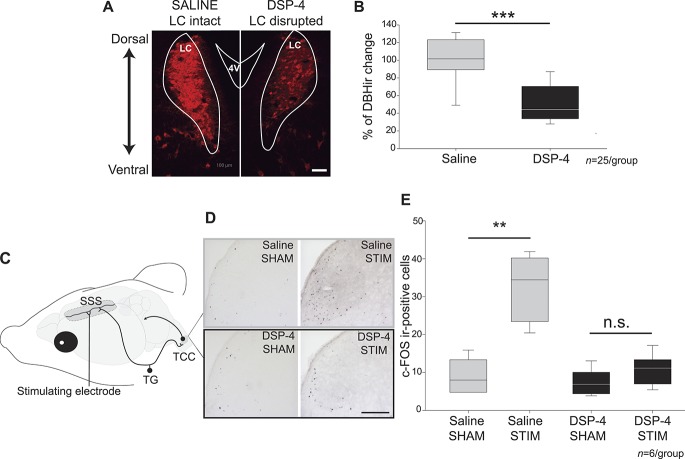
DSP-4 treatment chronically disrupts locus coeruleus (LC) noradrenergic projections and inhibits trigeminovascular activation. (A) Dopamine-β-hydroxylase (DBH) immunofluorescence in the LC in saline and DSP-4–treated animals (white scale bar = 100 µm, white outlines were extracted from rat brain atlas^38^). (B) Percentage change in LC area immunoreactive for DBH in saline (n = 25, gray box)- and DSP-4 (n = 25, black box)–treated animals normalized to controls. DSP-4 treatment induced a significant loss of noradrenergic cells in the LC to 54 ± 5% compared with saline-treated animals (*t*_(48)_ = 6.32, *P* < 0.001) (Data are expressed as median and interquartile ranges ***P = 0.000027). (C) Schematic representation of the experimental setup of nociceptive trigeminovascular activation as a model of migraine-related pain processing. A bipolar stimulating electrode was placed over the superior sagittal sinus (SSS) to induce dural-evoked activation of trigeminal afferents from the trigeminal ganglion (TG) that innervate the dural vasculature. Neuronal activation was analysed by c-Fos immunohistochemistry detection within the trigeminocervical complex (TCC). (D) Photomicrographs showing c-Fos immunohistochemistry expression in the TCC in a representative animal of each experimental group. (E) Number of c-Fos immunoreactive positive cells in the TCC (n = 6/group). Electrical stimulation induced a 4-fold increase in neuronal activation in the TCC compared with sham in saline-treated rats (*U* = 0, *P* = 0.006). However, in DSP-4–treated rats, there was no difference in neuronal activation between sham and stimulated animals (*U* = 7, *P* = 0.144) (Data are expressed as median and interquartile ranges, black scale bar = 200 µm, **P = 0.006). 4 V = fourth ventricle, DBHir = area immunoreactive for DBH, ir = immunoreactive, STIM = electrically stimulated.

Two weeks after a single intraperitoneal injection of DSP-4 (50 mg/kg), a well-established selective toxin for LC noradrenergic neurons that acts through noradrenaline transporter uptake mechanisms,^40^ we observed a significant reduction in noradrenergic neurons in the LC as identified by decreased DBH immunoreactivity, compared with saline-treated rats (n = 25/group, 54 ± 5% reduction; *t*_(48)_ = 6.32, *P* ≤ 0.0001; Figs. 2A and B). We also observed a significant reduction in DBH immunoreactive processes, most likely as a result of a loss of LC projection neurons in the cortex (n = 4/group, 92.9 ± 3.5% reduction; *t*_(6)_ = 14.15, *P* ≤ 0.0001) and upper spinal cord (n = 4/group, 90.2 ± 4.3% reduction; *t*_(6)_ = 2.58, *P* = 0.043) when comparing DSP-4 to saline-treated rats.

#### 3.1.3. Chronic disruption of the locus coeruleus inhibits dural-evoked trigeminovascular activation

Animals randomly assigned to receive either saline or DSP-4 (n = 12 each) were further subjected to sham or electrical stimulation (n = 6 per group) of the SSS to induce dural-evoked nociceptive activation of the trigeminovascular system (Fig. 2C). Neuronal activation was then analysed by immunohistochemical detection of the marker of neuronal activation c-Fos within the dorsal horn of the TCC (Fig. 2D).

Electrical stimulation of the SSS in saline-treated animals induced a significant 4-fold increase in neuronal activation in the TCC compared with sham-treated rats (*U* = 0, *P* = 0.006, Fig. 2E). However, in animals receiving DSP-4, where the LC was chronically disrupted, stimulation of the SSS failed to induce an increase in neuronal activation, showing similar levels of c-Fos expression as sham animals (*U* = 7, *P* = 0.144, Fig. 2E). Thus, both acute lesioning and chronic ablation of the LC inhibits dural-evoked trigeminovascular activation at the level of the TCC.

#### 3.1.4. α2-adrenoceptors–dependent inhibition of locus coeruleus activity decreases dural-evoked trigeminovascular activation

Given our demonstration of the antinociceptive effects of acute and chronic disruption of the LC, we next sought to explore the impact of its pharmacological modulation through adrenoceptors using in vivo electrophysiology of dural-evoked trigeminovascular activation in the TCC combined with local microinjection into the LC.

**Figure 3.**
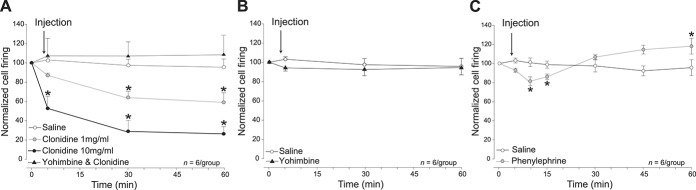
Trigeminovascular activation is modulated by locus coeruleus (LC) adrenoceptors. Time course changes in the average response of dural-evoked Aδ-fiber trigeminal neurons after microinjections in the LC. (A) Microinjection of the α2-adrenoceptor agonist clonidine induced a concentration-dependent reduction of the dural-evoked neuronal activation in the TCC when compared with the vehicle control group (F_4.45,66.8_ = 12.96, *P* < 0.0001), reaching a significant decrease at 30 minutes after injection of the lowest concentration (1 mg/mL, *t*_(12)_ = −2.72, *P* = 0.019) and at 5 minutes after injection of the highest concentration (10 mg/mL, *t*_(9)_ = −7.56, *P* < 0.001). Pretreatment with the α2-adrenoceptor antagonist yohimbine blocked the inhibitory effect (F_11,77_ = 0.88, *P* = 0.561). (B) Microinjection of the α2-adrenoceptor antagonist yohimbine alone had no significant effect on dural-evoked trigeminovascular activation in the TCC when compared with the vehicle control group (F_11,110_ = 0.44, *P* = 0.94). (C) Microinjection of α1-adrenoceptor agonist phenylephrine in the LC induced a biphasic response in dural-evoked activation in the TCC. This response was characterised by an initial significant reduction of the neuronal activation to 81% ± 5 that lasted 15 minutes (at 5 minutes: *t*_(10)_ = −2.61, *P* = 0.026; at 15 minutes: *t*_(10)_ = −2.54, *P* = 0.029), followed by a significant increase, reaching its maximum of 118% ± 8 at 60 minutes after injection (*t*_(10)_ = 3, *P* = 0.013). (Data are expressed as mean ± SEM, *P < 0.05). TCC, trigeminocervical complex.

In agreement with an inhibitory action on LC activity, the microinjection of the α2-adrenoceptor agonist clonidine in the LC significantly inhibited TCC activation in a dose-dependent manner (F_4.45,66.8_ = 12.96, *P* ≤ 0.0001, Fig. 3A), reaching a significant decrease at 30 minutes after injection of the lowest concentration (1 mg/mL, *t*_(12)_ = −2.72, *P* = 0.019) and at 5 minutes after injection of the highest concentration (10 mg/mL, *t*_(9)_ = −7.56, *P* ≤ 0.0001). An effect that was blocked when pretreating with the α2-adrenoceptor antagonist yohimbine (F_11,77_ = 0.88, *P* = 0.561, Fig. 3A). Yohimbine alone microinjected in the LC did not have an effect on dural-evoked responses in the TCC (F_11,110_ = 0.44, *P* = 0.94, Fig. 3B). There were no significant differences in baseline spontaneous firing throughout the entire experiment, suggesting the LC effects were nociceptive-specific (F_11,220_ = 0.92, *P* = 0.518).

#### 3.1.5. Locus coeruleus modulates dural-evoked trigeminovascular activation through α1-adrenoceptors

Given the inhibitory actions of α2-adrenoceptor activation and disruption of the LC, we sought to explore the impact of modulation of LC α1-adrenoceptors that has previously been reported to be pronociceptive.^39^ Microinjection of the α1-adrenoceptor agonist phenylephrine into the LC resulted in an acute reduction (81 ± 5%, *t*_(10)_ = −2.54, *P* = 0.029) in dural-evoked TCC neuronal activation that transitioned to increased dural-evoked TCC neuronal activation (118 ± 8%, *t*_(10)_ = 3.01, *P* = 0.013, Fig. 3C) over the 1-hour recording window. This is in agreement with a prolonged facilitation of nociceptive responses demonstrated by others.^39^ There were no significant differences in baseline spontaneous firing throughout the entire experiment, suggesting the LC effects were nociceptive-specific (F_1.49,14.89_ = 1.68, *P* = 0.105).

#### 3.1.6. Chronic disruption of the locus coeruleus increases susceptibility to cortical spreading depression

Given the impact of LC disruption on dural-evoked trigeminovascular activation, its previously identified role in cortical blood flow regulation,^23^ and the fact that it provides the only source of neocortical noradrenaline,^5,32,41^ we sought to explore the effect of LC disruption on CSD, the presumed underlying phenomenon of migraine aura.^3,4,52^

**Figure 4.**
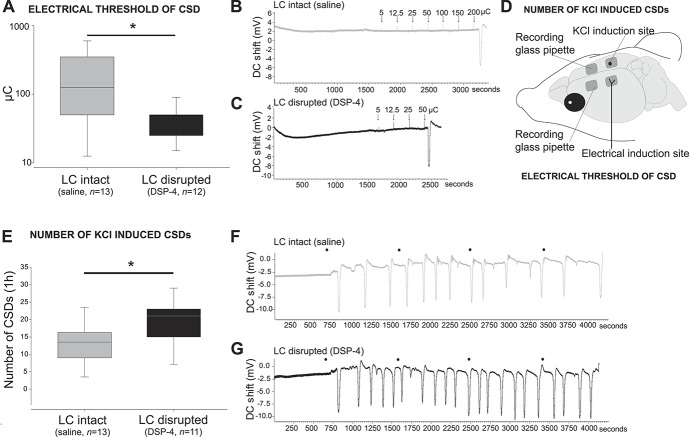
Chronic disruption of the locus coeruleus (LC) increases susceptibility to cortical spreading depression (CSD). (A) The electrical threshold (measured as the current intensity times stimulus duration; microCoulombs [µC]) to induce a CSD was significantly lower in the animals with their LC disrupted (*U* = 43, *P* = 0.036) indicating an increased susceptibility to CSD (Data are expressed as median and interquartile range in a logarithmic scale, *P < 0.05). In vivo cortical DC shift readings in an LC-intact (B) and an LC-disrupted (C) representative animal showing the application of increasing electric charges until a CSD was achieved. (D) Schematic representation of the experimental setup of CSD as a preclinical model of migraine aura. The electrical threshold to develop a CSD was determined on the left hemisphere by stimulating with a bipolar electrode increasing the electric charge. Once a CSD was induced, the number of KCl-induced CSDs was determined on the right hemisphere by placing a cotton ball soaked in 1-M KCl. (E) The number of KCl-induced CSDs was significantly higher in the animals with their LC disrupted (*t*_(23)_ = −2.5, *P* = 0.018) confirming an increased susceptibility to CSD (Data are expressed as mean ± SEM, *P* < 0.05). In vivo cortical direct current (DC) shift readings in an LC-intact (F) and an LC-disrupted (G) representative animal showing the application of a cotton ball soaked in 1-M KCl on the cortex surface (which was refreshed with 5 µL every 15 minutes, black circles) inducing multiple CSDs throughout the 1-hour recording.

A separate randomly assigned cohort of rats were injected with either saline or DSP-4 (n = 25) and studied to measure their CSD susceptibility. Cortical spreading depression can be induced with several methods; the most robust methods being electrical and chemical stimulation. Electrical stimulation allows for the determination of an individual CSD threshold, whereas chemical stimulation determines the number of CSDs that occur after the application of the stimulus. To reduce the number of animals used and in accordance with previous studies,^28^ we used the electrical method to determine the CSD threshold in the left hemisphere and subsequently the chemical method to determine the number of CSDs that occurred in response to repetitive KCl application over the right hemisphere (Fig. 4D).

Chronic disruption of the LC through DSP-4 treatment resulted in a significant decrease in the threshold required to induce CSD when compared with saline-treated rats, from 209 ± 58 µC to 43 ± 7 µC (*U* = 43, *P* = 0.036), indicating an increased susceptibility to CSD initiation (Figs. 4A–C). In agreement with an increased susceptibility to CSD, application of 1-M KCl over the cortex in DSP-4–treated rats resulted in a significant increase in the number of CSDs recorded over the 1-hour period from 13 ± 2 to 20 ± 2 (*t*_(23)_ = −2.5, *P* = 0.018, Figs. 4E–G) that was more prominent in the final 30 minutes of the recording (supplementary Figure 1, available at http://links.lww.com/PAIN/A682).

## 4. Discussion

Our experiments reveal a potent role for LC dysregulation in the modulation of dural-evoked trigeminovascular activation in the TCC and the susceptibility to CSD, the presumed underlying mechanism of migraine aura. Using 2 different validated preclinical models for migraine, we have identified that LC disruption decreases TCC activation to durovascular nociceptive stimulation. Importantly, activation of the LC has demonstrated divergent effects on pain, with both antinociceptive and pronociceptive effects.^14,27^ To confirm that it was reduced LC signalling that was responsible for the observed antinociceptive effects, we chronically ablated LC noradrenergic projections and recorded TCC dural-evoked responses 2 weeks later.

**Figure 5.**
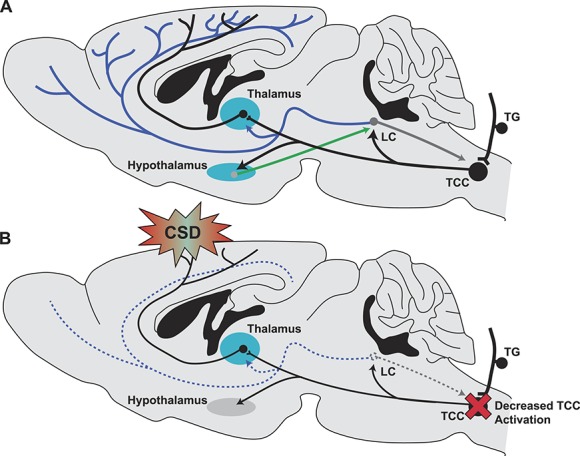
The locus coeruleus (LC) and migraine. The noradrenergic system plays a crucial role in the regulation of arousal and nociception. As one of the major sources of noradrenaline in the central nervous system (CNS), we sought to determine the role of the LC in migraine pathophysiology. (A) Trigeminal nociceptive input from the head is conveyed through primary afferents arising in the trigeminal ganglion (TG) that synapse centrally on the trigeminocervical complex (TCC). From the TCC, ascending projections largely target the sensory thalamic nuclei; however, excitatory projections also target the LC resulting in its activation. During wakefulness, the LC receives descending excitatory projections from the hypothalamus that promotes its activation (green arrow). In turn, the LC sends noradrenergic projections to most of the CNS, including descending projections to the TCC and spinal cord, as well as ascending projections to the thalamus and cortex, having roles in arousal and nociceptive processing. (B) We have demonstrated that loss of LC noradrenergic signalling inhibits trigeminal nociceptive signalling at the level of the TCC, while paradoxically increasing the susceptibility to cortical spreading depression (CSD). As such, we propose that inhibition of the LC exerts a potent antinociceptive effect on trigeminal nociceptive processing. As the LC shows diurnal activity, falling almost completely silent during sleep, we further hypothesise that sleep-induced LC inhibition may be a potential mechanism for sleep-induced migraine attack normalisation.

The observed decrease in trigeminovascular activation was mimicked by the α2-adrenoceptor agonist clonidine that has been shown to inhibit LC neuronal activity,^16,34^ suggesting that acute and chronic reductions in LC-derived noradrenergic signalling is antinociceptive at the level of the TCC. This is further supported by the facilitation of TCC dural-evoked trigeminal activation by the α1-adrenoceptor agonist phenylephrine that has previously been shown to be pronociceptive and excites LC neurons.^36,39^ Importantly, there was no significant alteration of background trigeminovascular activity suggesting that the observed effects were nociceptive-specific. As such, we hypothesise that the LC may be a key nucleus involved in sleep-induced migraine attack normalisation. Locus coeruleus activity is lowest during sleep,^30^ and as such, sleep-induced reductions in LC activity may act to inhibit aberrant nociceptive-specific trigeminal activity at the level of the TCC and reduce the sensory input to the perturbed migraine brain.^3^ Correspondingly, it is known that TCC neuronal excitability increases in migraine patients because they near the next attack.^47^ As such, it could be hypothesised that decreasing LC activity would act to normalise this developing excitability with subsequent impacts on LC arousal-related mechanisms (Fig. 5), leading to abnormal fatigue.^21,22^ As discussed, the LC has been shown to have divergent effects on spinal nociception, with both pronociceptive and antinociceptive effects.^14,27^ This is likely due to the heterogeneous population of LC neuronal projections with both descending and ascending pathways.^27^ As we recorded wide dynamic range neuronal responses in the TCC as a surrogate marker of trigeminal mediated nociceptive activation, we are unable to determine whether LC ablation or pharmacological modulation impacts pain behaviours in conscious freely behaving animals. Future studies should determine the specific effects of targeting ascending and descending pathways individually in such conditions.

Given that LC ablation with DSP-4 depleted cortical and spinal noradrenergic expression, we hypothesised that loss of ascending noradrenergic signalling to the cortex would render it hyperexcitable and decrease the threshold for CSD. This is in agreement with the ability of LC stimulation to alter cortical blood flow.^23^ Chronic ablation of the LC resulted in a significant decrease in the electrical threshold required to induce CSD and a significant increase in the number of CSDs induced by repetitive KCl application to the cortex. Our results suggest that dysregulation of the LC may increase the likelihood of CSD induction and migraine aura. This is in agreement with a previous study where indirect activation of the LC through vagus nerve stimulation^10,13^ decreased the susceptibility to CSD.^9^ Importantly, there remains some controversy over the impact of LC ablation on cortical extracellular noradrenaline levels^40^ because DSP-4–treated animals have been shown to have unchanged or increased extracellular concentrations in the cortex despite decreased tissue levels.^6^ This increase is believed to result from passive spread from noncoerulean sources combined with a lack of local noradrenaline uptake. As such, local tissue decreases may occur in the presence of an increased extracellular concentration of noradrenaline that may augment CSD susceptibility.

Taken together, our results show a direct involvement of the LC in the regulation of migraine pathophysiology, whereby dysfunctional LC signalling may alter attack susceptibility. Although the existing models cannot reproduce the intricate modulation that the LC exerts throughout the central nervous system, we determined that decreased spinal LC noradrenergic signalling inhibits dural-evoked trigeminal activation at the level of the TCC. This highlights a potential antinociceptive action of LC inhibition on migraine-related pain. Conversely, decreased cortical LC noradrenergic signalling reduced the threshold for CSD induction, suggesting the potential for LC dysregulation to impact the occurrence of migraine aura. Our results highlight the LC and its noradrenergic projections as a key nucleus in the regulation of migraine pathophysiology. Given the arousal-related functions of the LC, its role in stress regulation and cognition, we further predict that the dysfunctional LC signalling may in part underlie migraine-associated symptoms including abnormal fatigue.

## Conflict of interest statement

M. Vila-Pueyo, L.C. Strother, and M. Kefel declare no competing financial interests. P.J. Goadsby reports, unrelated to the current project, grants, and personal fees from Allergan, Amgen, and Eli-Lilly and Company; and personal fees from Akita Biomedical, Alder Biopharmaceuticals, Cipla Ltd, Dr Reddy's Laboratories, eNeura, Electrocore LLC, Novartis, Pfizer Inc, Quest Diagnostics, Scion, Teva Pharmaceuticals, Trigemina Inc, Scion; and personal fees from MedicoLegal work, Journal Watch, Up-to-Date, Massachusetts Medical Society, Oxford University Press; and in addition, P.J. Goadsby has a patent Magnetic stimulation for headache assigned to eNeura without fee. P.R. Holland reports, unrelated to the current project, honoraria for educational and advisory purposes from Allergan, Novartis and TEVA as well as research funding from Amgen and Eli-Lilly.

## Supplementary Material

SUPPLEMENTARY MATERIAL

## Appendix A. Supplemental digital content

Supplemental digital content associated with this article can be found online at http://links.lww.com/PAIN/A682.

## Supplemental video content

Video content associated with this article can be found at http://links.lww.com/PAIN/A683.
