# Supplementary material for: Divergent influences of the locus coeruleus on migraine pathophysiology
Source: Pain. 2018 Dec 14;160(2):385–94. doi: 10.1097/j.pain.0000000000001421 (PMC6343946; doi:10.1097/j.pain.0000000000001421)
Supplement: SUPPLEMENTARY MATERIAL [file jop-160-385-s001.docx]

**Supplementary Figure 1. Chronic disruption of the locus coeruleus increases susceptibility to KCl-induced cortical spreading depression.** A raster plot of the temporal occurrence of cortical spreading depressions (CSDs; black dots) in saline treated, locus coeruleus (LC) intact (top), compared to DSP-4 treated LC disrupted rats. There was a significant increase in the number of KCl-induced CSDs in the animals with their LC disrupted (*t*_(23)_ = -2.5, *p* = 0.018).
